# Molecular Cloning and Gene Expression Analysis of the Leptin Receptor in the Chinese Mitten Crab *Eriocheir sinensis*

**DOI:** 10.1371/journal.pone.0011175

**Published:** 2010-06-17

**Authors:** Hui Jiang, Fei Ren, Jiangling Sun, Lin He, Weiwei Li, Yannan Xie, Qun Wang

**Affiliations:** Department of Biology, East China Normal University, Shanghai, China; Baylor College of Medicine, United States of America

## Abstract

**Background:**

Leptin is an adipocyte-derived hormone with multiple functions that regulates energy homeostasis and reproductive functions. Increased knowledge of leptin receptor function will enhance our understanding of the physiological roles of leptin in animals.

**Methodology/Principal Findings:**

In the present study, a full-length leptin receptor (*lepr*) cDNA, consisting of 1,353 nucleotides, was cloned from Chinese mitten crab (*Eriocheir sinensis*) using rapid amplification of cDNA ends (RACE) following the identification of a single expressed sequence tag (EST) clone in a cDNA library. The *lepr* cDNA consisted of a 22-nucleotide 5′-untranslated region (5′ UTR), a 402-nucleotide open reading frame (ORF) and a 929-nucleotide 3′ UTR. Multiple sequence alignments revealed that Chinese mitten crab *lepr* shared a conserved vacuolar protein sorting 55 (Vps55) domain with other species. Chinese mitten crab *lepr* expression was determined in various tissues and at three different reproductive stages using quantitative real-time RT-PCR. *Lepr* expression was highest in the intestine, thoracic ganglia, gonad, and accessory gonad, moderate in hepatopancreas and cranial ganglia, and low in muscle, gill, heart, haemocytes, and stomach. Furthermore, *lepr* expression was significantly higher in the intestine, gonad and thoracic ganglia in immature crabs relative to precocious and mature crabs. In contrast, *lepr* expression was significantly lower in the hepatopancreas of immature crabs relative to mature crabs.

**Conclusions/Significance:**

We are the first to identify the *lepr* gene and to determine its gene expression patterns in various tissues and at three different reproductive stages in Chinese mitten crab. Taken together, our results suggest that *lepr* may be involved in the nutritional regulation of metabolism and reproduction in Chinese mitten crabs.

## Introduction

Leptin is multifunctional hormone synthesized primarily by adipocytes [1]. Leptin is involved not only in the control of food intake, energy expenditure and metabolism, but also in the regulation of reproduction [2], [3]. Thus, leptin may act as a critical link between adipose tissue and the neuroendocrine axis, determining whether existing fat energy reserves are sufficient for normal reproductive function [4], [5]. Leptin influences satiety and adiposity and is associated with the regulation of puberty onset, fertility and pregnancy in a variety of species [6]. However, increasing evidence indicates that leptin is produced in many tissues other than adipocytes, and that enhanced leptin levels are associated with the advent of reproductive maturity and fertility [7], [8], including gonad maturation and embryonic development [9].

Cognate receptors for leptin have been identified in gonadal and non-gonadal tissues in all species studied to date [10]. The leptin receptor is single transmembrane glycoprotein with homology to interleukin 6 that belongs to the class I cytokine receptor superfamily [11]. Isoforms of leptin receptors with cytoplasmic regions of varying lengths due to alternative splicing or degradation have been reported [12].

The Chinese mitten crab *(Eriocheir sinensis)* (Henri Milne Edwards 1854) is a unique species of crab that is native to China. It is a catadromous crustacean with a life span of approximately two years. The crab enters the reproductive season in its second year and dies shortly after completing reproduction [13], [14], [15], [16], [17]. Various problems, including sexual precosity and diseases, caused by bacteria, viruses, or rickettsia-like organisms have been reported in cultured Chinese mitten crab populations since the development of intensive aquaculture in early 1980s [18], [19]. Precocious crabs mature and die early at a small size that leads to catastrophic losses for farmers and seriously restricts the development of crab aquaculture [20]. The molecular mechanisms underlying Chinese mitten crab sexual precosity remain unclear [21].

In our previous studies to identify molecular signaling pathways involved in Chinese mitten crab reproduction, we constructed expressed sequence tag (EST) cDNA libraries from hepatopancreas and testis [19], [22], and analyzed differentially expressed genes in the accessory sex gland using suppression subtractive hybridization (SSH). We identified a partial EST clone from the hepatopancreas library in Chinese mitten crab that shares high sequence identity to *lepr* gene from other species [19]. This was the first report of a crustacean *lepr* gene, which may regulate male reproduction in *E. sinensis*, similar to its role in other animals.

The main objectives of the present study included: (1) to isolate the full-length *E. sinensis lepr* cDNA, (2) to determine the genetic structure of *lepr*, (3) to determine evolutionary status of *E. sinensis lepr*, (4) to determine the expression patterns of *lepr* in different tissues, and (5) to examine *lepr* expression in normal and precocious crabs.

## Materials and Methods

### Tissue Collection

Chinese mitten crabs at three different reproductive stages were purchased from the Tongchuan Road aquatic product market, Shanghai, China. Healthy immature (in the rapid developmental stage) and precocious Chinese mitten crabs were obtained in July, and normal mature Chinese mitten crabs were obtained in October. The crabs were placed in an ice bath for 1–2 min, until they were lightly anesthetized. Various Chinese mitten crab tissues (muscle, heart, stomach, haemocytes, hepatopancreas, gonad, accessory gonad, cranial ganglia, thoracic ganglia, gill and intestine) were dissected, immediately frozen in liquid nitrogen and stored at −80°C until RNA and DNA were extracted.

### cDNA Library Construction and EST Analysis

A cDNA library was constructed from the hepatopancreas of *E. Sinensis*. Random sequencing of the library using a T3 primer yielded 3,355 successful sequencing reactions. BLASTx analysis of all of the EST sequences showed that a single EST (EST No. FG359007; length: 705 bp) was homologous to the *Rhodnius prolixus lepr* gene (AAQ20841), and it was used to clone the full-length *lepr* cDNA from *E. sinensis*.

### Cloning the Full-Length *E. Sinensis lepr* cDNA

A total of 3,297 high quality ESTs were generated from the sequencing of a Chinese mitten crab cDNA library. BLAST analysis showed that four ESTs were homologous to the previously identified *lepr*, which could be assembled into a 705 bp singlet. Individual clones corresponding to the four ESTs were selected for complete sequencing with a T7 primer (Table 1). Two gene-specific primers, GSP1 and GSP2 (Table 1), were designed based on the 705 bp singlet to clone the 5′ and 3′ ends of the *lepr* cDNA by rapid amplification of cDNA ends (RACE) using the SMART^TM^ RACE cDNA Amplification Kit (Clontech, USA). The cDNA sequences generated from the corresponding clones and RACE amplification were assembled into the full-length cDNA. Finally, a pair of gene-specific primers, LR-S and LR-R (Table 1), was designed to amplify the full-length cDNA to verify the sequence.

**Table 1:** Oligonucleotide primers used in the experiments.

Primer name	Sequence (5′-3′)
T3	AATTAACCCTCACTAAAGGG
T7	TAATACGACTCACTATAGG
GSP1	AACACCCTCAGCAGTGCCCTTTATC
GSP2	GCCGCCATCGTTGTCTCTGCTTTTG
LR-S	GGAGTTGGCATACTTCGT
LR-R	TTAGGGTTTGGGAGTTTGT
5′SP1	CTGGCTGACACTTTGGCGAATC
5′SP2	GAGTGGGTCTGTCCCGATGTAAT
5′SP3	GCCAAAAAGAGGAAGGTGAGG
3′SP1	TGTGATAAAGGGCACTGCTGAG
3′SP2	AGGAGTAGTGTGTAACAGAGGGCAT
3′SP3	CACAAGATGCCTGCTTACCCCT
rtLR-S	GGTGACCCTTGCCTTCGC
rtLR-R	TGCCGCCTGTGTCCTCTG
Actin-S	CTCCTGCTTGCTGATCCACATC
Actin-R	GCATCCACGAGACCACTTACA

### Isolating the *E. Sinensis lepr* Genomic Clone

Genomic DNA was extracted from Chinese mitten crab hepatopancreas using the Axyprep™ Multisource Genomic DNA Miniprep Kit (Axygen, USA) according to the manufacturer's instructions. To obtain the sequence of the *lepr* genomic DNA clone, gene-specific primers, LR-S and LR-R (Table 1), were designed based on the cDNA sequence. The 5′ and 3′ untranslated regions (UTRs) were cloned using the Genome Walking Kit (TaKaRa, Dalian, China) according to manufacturer's instructions. In these reactions, specific primers, 5′SP1, 5′SP2, 5′SP3, 3′SP1, 3′SP2 and 3′SP3 (Table 1), were designed to amplify the *lepr* 5′ and 3′ UTRs. The resulting products were sequenced with a 3730XL DNA analyzer (Applied Biosystems, Foster City, CA) according to manufacturer's protocol. The sequences were assembled using CAP3 (https://www.genome.clemson.edu/cgi-bin/cugi_cap3?advanced=1).

### Sequence Analysis, Multiple Sequences Alignment, and Phylogenetic Analysis

Sequence similarity analyses were performed using the Blast program at the National Center for Biotechnology Information (http://www.ncbi.nlm.nih.gov/blast). The open reading frame (ORF) for the *lepr* cDNA was determined using ORF Finder (www.ncbi.nlm.nih.gov/gorf/) and translated into the corresponding amino acid sequence. The predicted signal peptide sequence was identified using the Signal 3.0 Server (www.cbs.dtu.dk/services/SignalP). Protein motif features were predicted using the Simple Modular Architecture Research Tool (SMART) (http://www.smart.emblheidelberg.de). Exon and intron positions were confirmed using the Spidey-mRNA-to-genomic alignment program (http://www.ncbi.nlm.nih.gov/IEB/Research/Ostell/Spidey/) [23], [24]. Sequence alignments were determined using the ClustalX software package. The sequences derived from the nr database of the NCBI are represented using their official names. The protein sequences were aligned using the Clustal program [25]. Phylogenetic analyses and statistical neighbour-joining bootstrap tests of the phylogenies were carried out using the Mega package [26].

### RT-qPCR Analysis of mRNA Expression

The mRNA expression patterns of *lepr* in different tissues (muscle, heart, stomach, haemocytes, hepatopancreas, gonad, accessory gonad, cranial ganglia, thoracic ganglia, gill and intestine) of healthy immature crabs (in the rapid developmental stage) and in six tissues (hepatopancreas, gonad, accessory gonad, cranial ganglia, thoracic ganglia and intestine) of healthy immature crabs, precocious crabs and normal mature crabs were determined by real-time RT-PCR [27]. First-strand cDNA synthesis and SYBR Green RT-PCR assays were performed according to the manufacturer's instructions. A pair of *lepr* specific primers, rtLR-S and rtLR-R (Table 1), were used to amplify a product of 164 bp, and a pair of β-actin primers, Actin-S and Actin-R (Table 1), were used to amplify a 266 bp fragment as the internal control. As a negative control, DEPC-treated water was substituted for the cDNA template. The SYBR Green RT-PCR assay was carried out using the CFX Manager TM System (Bio-Rad, USA). The real-time RT-PCR amplifications were carried out in a total volume of 25 µl containing 12.5 µl iQ SYBR Green SuPermix (Bio-Rad, USA), 0.5 µl diluted cDNA, 11.0 µl sterile water and 0.5 µl of each primer. The PCR temperature profile was 95°C for 3 min, followed by 40 cycles of 95°C for 10 s, 55°C for 30 s, and 95°C for 10 s.

Data from three replicate qRT-PCR samples were analyzed using CFX Manager TM software (Version 1.0). The comparative Ct method was used to analyze *lepr* expression levels. The ΔCt (differences in the Ct value between the target and internal control) for each sample was subtracted from that of the calibrator, which was called ΔΔCt, *lepr* expression levels were calculated using 2^−ΔΔCt^ and the value represented an n-fold difference relative to the control. The expression levels were imported to Microsoft Excel for subsequent data analyses. All data are presented as mean ± S.E. of relative mRNA expression. The results were subjected to t-tests, and differences were considered statistically significant at P<0.05.

## Results

### cDNA Cloning and Sequence Analysis of *E. sinensis lepr*

**Figure 1:**
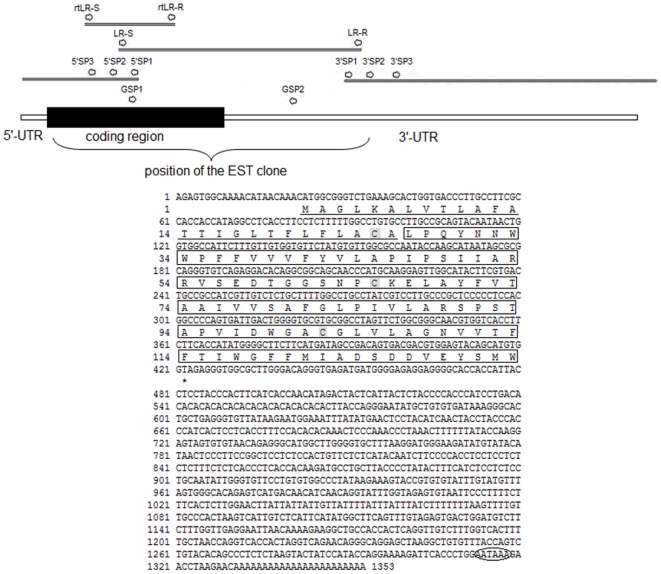
Nucleotide sequence and deduced amino acid sequence of Chinese mitten crab *lepr*. Nucleotides (upper) are numbered beginning at the 5′ end. Amino acids (lower), shown using one-letter abbreviations, are numbered beginning at the initiating methionine. The signal peptide is underlined, and the mature peptide is enclosed in the black box. The three conserved cysteine residues in the deduced amino acid sequence are shown in grey boxes. The stop codon is marked by an asterisk. The polyadenylation signal (AATAAA) is enclosed in the black ellipse. Arrows indicate the positions of primers; black boxes indicate the coding region; white lines indicate 5′ and 3′ UTRs; curly bracket indicate position of the EST clone. The *E. sinensis lepr* sequence was submitted to GenBank under accession number GU443952.

The full-length *E. sinensis lepr* cDNA was cloned by RT-PCR and RACE. The full-length of the *lepr* cDNA is 1,353 nucleotides in length and consists of a 22 nucleotide 5′ UTR, a 929 nucleotide 3′ UTR with a canonical polyadenylation signal site (AATAAA) and a polyA tail of 23 nucleotides. The *lepr* cDNA contains a 402 nucleotide open reading frame (ORF) encoding a putative polypeptide of 133 amino acids with a predicted molecular weight of 14.42 kDa and a theoretical isoelectric point of 4.46. The predicted lepr N terminus has features consistent with a signal peptide, as defined by SignalP program analysis (http://www.cbs.dtu.dk/services/SignalP/). The deduced polypeptide consists of a 107 amino acid mature peptide and a 26 amino acid signal peptide (Fig. 1) with a predicted cleavage site between amino acid positions 26 and 27 (ACA/LP).

### Genomic Cloning and Genetic Structure of *E. sinensis lepr*

**Figure 2:**
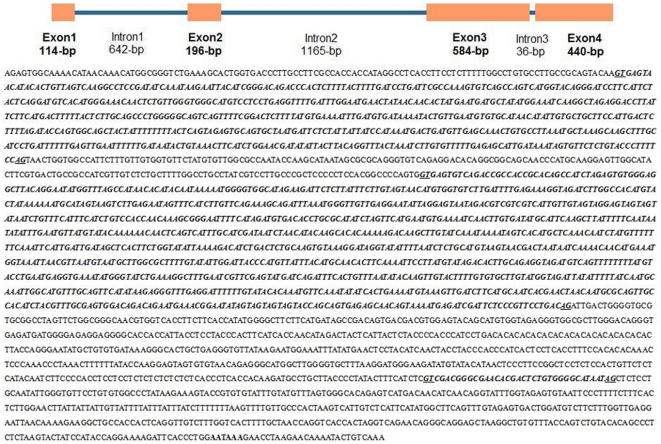
Genomic organization and nucleotide sequence of Chinese mitten crab *lepr*. *Lepr* intron sequences are shown in bold and italics. Intron dinucleotide acceptor and donor sites for RNA splicing are underlined. The polyadenylation signal is shown in bold.

The genomic clone of *E. sinensis lepr* consists of 3,880 bp and is comprised of four exons interrupted by three introns (Fig. 2). The *lepr* genomic sequence appears to contain typical intron-exon junction structures with donor and acceptor (GT and AG, respectively) dinucleotide sequences similar to those found in most vertebrate and invertebrate splice sites. Furthermore, the *lepr* genomic sequence was in good agreement with its cDNA sequence.

### Homology Analysis of *E. sinensis lepr*

**Figure 3:**
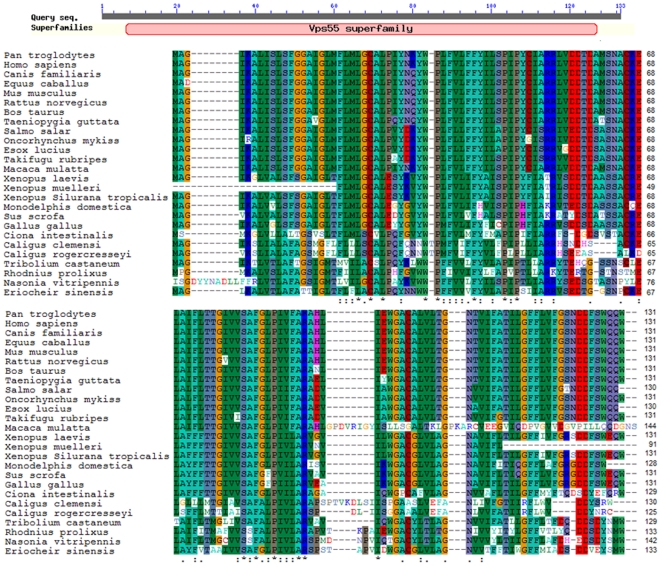
ClustalX alignment of *lepr* from *E. sinensis* and 25 other organisms. The vacuolar protein sorting 55 (Vps55) domain in *lepr* starts at Leu7 and ends at Asp127.

Alignment of *E. sinensis lepr* with other *lepr* amino acid sequences from vertebrates and invertebrates showed they share similarities and conserved amino acid sequences (Table 2). Multiple sequence alignment (Fig. 3) revealed that three cysteine residues (Cys25, Cys65 and Cys102) were highly conserved. SMART program analysis revealed that the *E. sinensis lepr*-encoded polypeptide contains a conserved domain and amino acid residues critical for its fundamental structure and function, including the vacuolar protein sorting 55 (Vps55) domain. The Vps55 domain in the *lepr* protein was comprised of 120 amino acids, starting at position Leu7 and ending at position Asp127.

**Table 2:** *Lepr* sequences identities between *E. sinensis* and 25 other organisms.

Matched species	Accession No.	%Identity
*Xenopus laevis*	NP_001080834	65%
*Nasonia vitripennis*	XP_001605479	64%
*Macaca mulatta*	XP_001082721	63%
*Xenopus muelleri*	ACB72435	61%
*Tribolium castaneum*	XP_973202	61%
*Takifugu rubripes*	NP_001129622	60%
*Esox lucius*	ACO13198	60%
*Salmo salar*	NP_001139931	60%
*Oncorhynchus mykiss*	ACO08366	59%
*Xenopus (Silurana) tropicalis*	NP_001017102	59%
*Monodelphis domestica*	XP_001364832	59%
*Pan troglodytes*	XP_528101	58%
*Canis familiaris*	XP_532817	58%
*Mus musculus*	NP_080885	58%
*Bos Taurus*	NP_001029913	58%
*Taeniopygia guttata*	XP_002198079	58%
*Gallus gallus*	NP_001007959	58%
*Equus caballus*	XP_001495850	57%
*Rattus norvegicus*	NP_064484	57%
*Homo sapiens*	NP_059996	56%
*Sus scrofa*	NP_001138860	56%
*Rhodnius prolixus*	AAQ20841	56%
*Ciona intestinalis*	XP_002128678	54%
*Caligus clemensi*	ACO14858	50%
*Caligus rogercresseyi*	ACO11244	50%

### Phylogenetic Analysis of *E. sinensis lepr*

**Figure 4:**
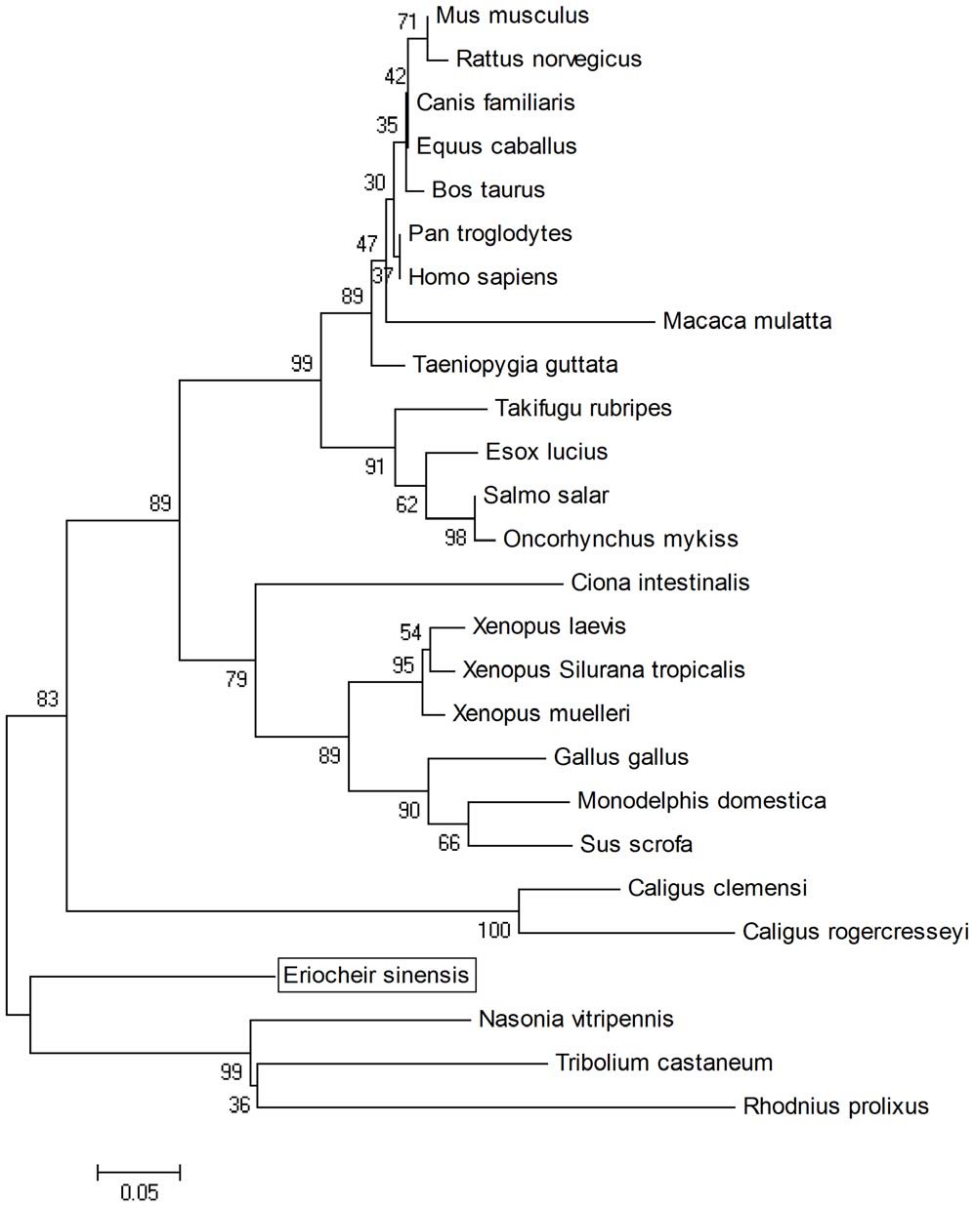
Phylogenetic tree of *lepr* amino acid sequences in four vertebrates and 22 invertebrates using the neighbour-joining method. The numbers in the phylogram nodes indicate percent bootstrap support for the phylogeny. The bar at the bottom indicates 5% amino acid divergence in sequences.

To evaluate the molecular evolutionary relationships between *E. sinensis lepr* and other *lepr* family members, a phylogenetic tree was constructed based on amino acid sequences using the neighbor-joining method (Fig. 4). There were two main branches with a strong bootstrap in the phylogenetic tree: the invertebrate *lepr* family members were clustered into a branch separate from the vertebrate branch. The results of phylogenetic analysis were in good agreement with the concept of traditional taxonomy. Numerous vertebrate *lepr* genes have been identified, however relatively few invertebrate *lepr* sequences are known; thus, the vertebrate branch is much more expansive than invertebrate branch. In the invertebrate branch, *E. sinensis* formed one monophyletic sub-branch, and the other three invertebrate species (*Nasonia vitripennis*, *Tribolium castaneum* and *Rhodnius prolixus*) formed a strong clade with bootstrap support.

**Figure 5:**
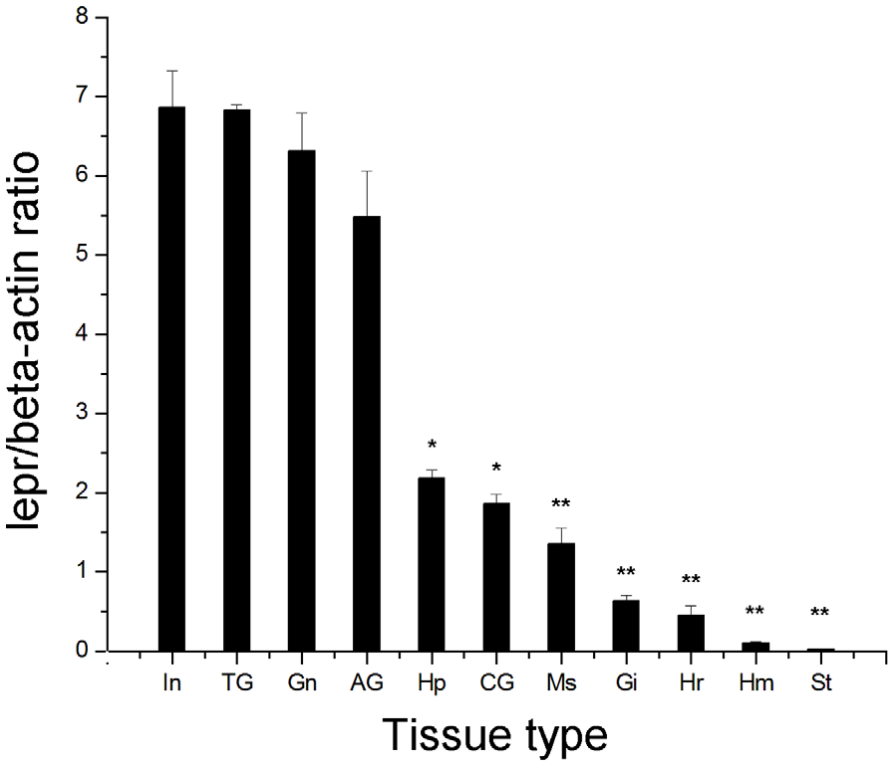
Tissue distribution of the *lepr* transcript measured by SYBR Green RT-PCR. Quantitative analysis of *lepr* gene expression relative to β-actin expression in different mitten crab tissues. Lepr gene expression was analyzed in the following tissues: In – intestine, TG – thoracic ganglia, Gn – gonad, AG – accessory gonad, Hp – hepatopancreas, CG – cranial ganglia, Ms – muscle, Gi – gill, Hr – heart, Hm – haemocytes and St – stomach. Expression data for each tissue were analyzed from three individual crabs. Vertical bars represent the mean ± S.E. (n = 3). Significant differences relative to controls are indicated with an asterisk (P<0.05) or with two asterisks (P<0.01).

### Tissue distribution of *E. sinensis lepr* Expression

Quantitative real-time RT-PCR was employed to investigate the distribution of *lepr* expression in different tissues from healthy immature crabs (in the rapid developmental stage) using β-actin as an internal control. The tissues examined included: intestine, thoracic ganglia, gonad, accessory gonad, hepatopancreas, cranial ganglia, muscle, gill, heart, haemocytes and stomach (Fig. 5). The results of these experiments showed that *lepr* was expressed in all of the tissues examined, but the expression levels varied between tissues. *Lepr* expression levels were highest in intestine, thoracic ganglia, gonad and accessory gonad, moderate in hepatopancreas and cranial ganglia, and only trace levels of expression were detected in muscle, gill, heart, haemocytes and stomach.

The highest *lepr* expression levels were detected in intestine, which were slightly higher than that of the thoracic ganglia, gonad and accessory gonad (P>0.05); a significant difference was found when *lepr* expression levels in intestine were compared with the expression levels in the hepatopancreas and cranial ganglia (P<0.05). The expression levels of *lepr* in muscle, gill, heart, haemocytes and stomach were significantly lower than the expression levels in intestine (P<0.01).

### Quantitative Analysis of *E. sinensis lepr* mRNA Expression

**Figure 6:**
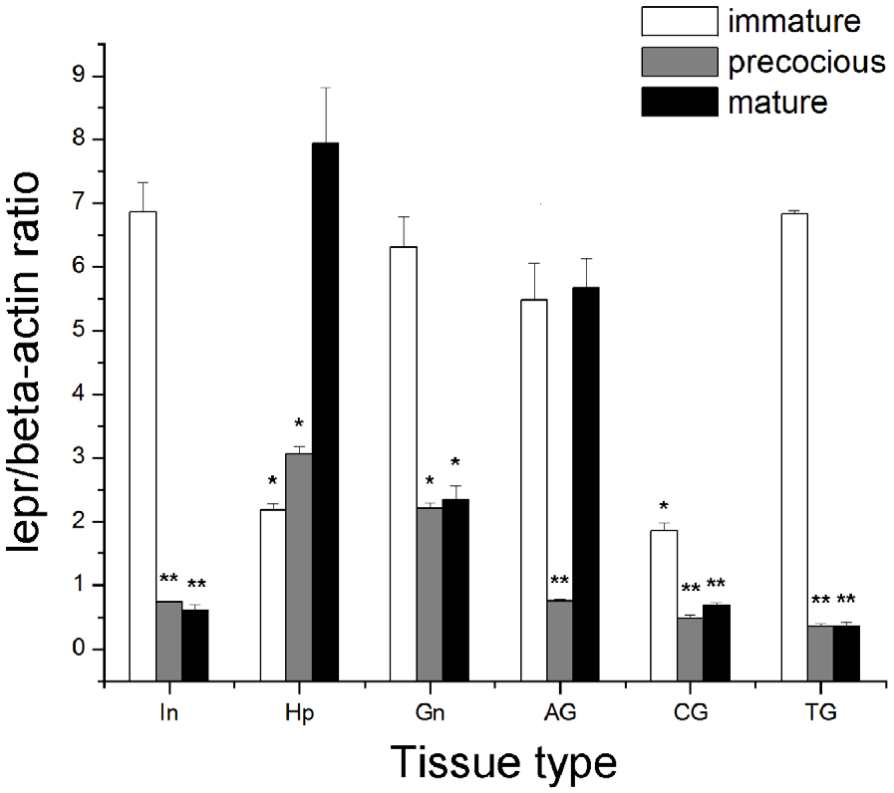
*Lepr* mRNA expression levels in six tissues from normal crabs (in the rapid developmental stage), precocious crabs and normal mature crabs. Values are shown as mean ± S.E. (n = 3). Significant differences relative to intestine are indicated with one asterisk (P<0.05) or with two asterisks (P<0.01).

The mRNA expression levels of *lepr* in six tissues (hepatopancreas, gonad, accessory gonad, cranial ganglia, thoracic ganglia and intestine) from healthy immature crabs (in the rapid developmental stage), precocious crabs and normal mature crabs was also measured by real-time RT-PCR using β-actin as an internal control (Fig. 6). The *lepr* mRNA expression levels in precocious crabs were relatively abundant in the hepatopancreas and gonad, and were lower in the intestine, accessory gonad, thoracic ganglia and cranial ganglia. However, *lepr* expression levels in normal mature crabs were highest in the hepatopancreas and accessory gonad, relatively abundant in gonad and much lower in the intestine, thoracic ganglia and cranial ganglia. The highest *lepr* expression levels were detected in the hepatopancreas of normal mature crabs.

The expression levels of *lepr* in the intestine and thoracic ganglia were significantly higher in healthy immature crabs than in precocious and normal mature crabs (P<0.01), and the same was true for *lepr* expression in the gonad and cranial ganglia (P<0.05). However, *lepr* expression in the hepatopancreas was significantly higher in normal mature crabs than in healthy immature and precocious crabs (P<0.05). Finally, *lepr* expression in the accessory gonad was higher in healthy immature and normal mature crabs than in precocious crabs (P<0.01).

## Discussion

Leptin and leptin receptors play key roles in energy homeostasis regulation and reproductive functions. An increased understanding of the function(s) of *E. sinensis lepr* may be beneficial with regard to controlling sexual precosity, developing strategies for health management of crab farming and elucidating regulatory mechanisms associated with reproduction. To date, many *lepr* genes has been identified and functionally characterized in vertebrates, however relatively few molecular or functional studies of invertebrate *lepr* genes have been conducted.

In the present study, the full-length *lepr* cDNA was cloned from *E. sinensis* and shown to encode a predicted polypeptide of 133 amino acids with a theoretical mass of 14.42 kDa and an isoelectric point of 4.46. Consistent with other known leptin receptor sequences, it contained a predicted signal peptide consisting of 26 amino acid residues. It also contained three conserved cysteine residues (Cys25, Cys65 and Cys102) that were identical to those in other leptin receptors [28], [29]. Multiple alignments revealed the conservation of the Vps55 domain among *lepr*-encoded proteins.

In the present study, *E. sinensis lepr* transcripts were detected in all tissues examined at different levels; it is notable that gene expression levels in healthy immature crabs (in the rapid developmental stage) were highest in the intestine, thoracic ganglia, gonad, accessory gonad and hepatopancreas. Notably, the intestine and hepatopancreas are involved in the final steps of food digestion and nutrient absorption [30]. In contrast, the *E. sinensis* gonad and accessory gonad are important components of the reproductive system [31], [32]. Furthermore, cranial ganglia and thoracic ganglia are important endocrine glands in *E. sinensis*, which secrete a variety of hormones that regulate the reproductive process [33], [34]. To a large extent, reproductive development is regulated by nutrition in *E. sinensis* [19]. During the rapid developmental stage in crabs, a significant amount of energy is stored in the hepatopancreas in preparation for the large energy output required during reproduction [35]. Taken together, the tissue distribution of *lepr* expression in healthy immature crabs suggests a role in nutritional metabolism and reproduction.

Expression levels of *lepr* were significantly different in six tissues between immature, precocious and mature crabs. *Lepr* expression levels in the intestine were highest in immature crabs, suggesting a role for *lepr* in nutrient absorption in developing crabs. The hepatopancreas is a sensitive indicator of lipid and carbohydrate metabolism and nutritional status in a variety of crustaceans [30]. Mature crabs showed the highest hepatopancreas levels of *lepr* expression, whereas immature crabs showed the lowest hepatopancreas levels of *lepr* expression. The different levels of *lepr* expression in the hepatopancreas of immature and mature crabs are consistent with the developmental status and nutritional requirements during these different stages. Taken together, these results suggest that the primary function of *lepr* in the digestive system may be associated with nutrient metabolism.

The cranial and thoracic ganglia are important endocrine glands in *E. sinensis* that secrete hormones involved in the regulation of the reproductive process during development [34]. In mature crabs, reproductive development is complete. *Lepr* expression levels were significantly higher in the cranial and thoracic ganglia of immature crabs than in mature crabs. Furthermore, in immature crabs, *lepr* expression was significantly higher in thoracic ganglia than in cranial ganglia. These results suggest that the role of *lepr* in thoracic and cranial ganglia may be associated primarily with the regulation of reproduction.

The development of the reproductive system in male Chinese mitten crabs includes two steps: development of the gonad (generally from June to August in the second year) [36], followed by development of the accessory sex gland (generally from August to November in the second year) [37], [38]. In immature crabs, the gonad is developing rapidly, whereas in mature crabs, the gonad has already fully developed and the accessory sex gland is developing rapidly. In the gonad, *lepr* expression levels were significantly higher in immature crabs than in mature and precocious crabs; this was in agreement with the development stage of the gonad. The accessory sex gland, which is an important component of the male reproductive system, plays an influential role during male reproduction [37] and it requires more nutrients than the testis during development. In the accessory sex gland, *lepr* expression levels were higher in immature and mature crabs than in precocious crabs; these differences may be related to accessory sex gland development. In mature crabs, the accessory sex gland is developing rapidly and may require the transport of additional nutrients from the hepatopancreas. In immature crabs, accessory sex gland development is initiated. In precocious crabs, *lepr* expression levels were low in both hepatopancreas and accessory sex gland tissues, suggesting that accessory sex gland development may depend on nutrient accumulation in the hepatopancreas, and that insufficient nutrient accumulation in the hepatopancreas may in turn affect accessory sex gland development. Taken together, these findings suggest that *lepr* may play a key role in the nutritional regulation of reproduction during the period of rapid development in *E. sinensis*.

To our knowledge, we are the first to report the sequence of the *lepr* gene and its expression patterns in *E. sinensis*. The results of the current study suggest that *lepr* may play a critical role in the regulation of reproductive maturity in *E. sinensis*. However, additional studies will be required to explore the underlying molecular mechanisms associated with sexual precocity. Furthermore, differentially spliced isoforms of the *lepr* gene may carry out distinct signaling functions. Future studies will clarify the role(s) of different *lepr* isoforms and uncover the specific signaling pathways that mediate the advent of reproductive maturity and fertility in *E. sinensis*.
